# Liver Transplant After Neoadjuvant Treatment for Long-Term Survivors With Intrahepatic Cholangiocarcinoma: Does It Have a Role?

**DOI:** 10.7759/cureus.75935

**Published:** 2024-12-18

**Authors:** Carina Teixeira, Bárbara Viamonte, Luís Graça, Hugo Pinto Marques, Inês Rego, Maria João Ribeiro

**Affiliations:** 1 Medical Oncology, Centro Hospitalar Universitário São João, Porto, PRT; 2 Radiology, Centro Hospitalar Universitário São João, Porto, PRT; 3 General Surgery, Centro Hospitalar Universitário São João, Porto, PRT; 4 Hepato-Biliopancreatic and Transplantation Centre, Hospital Curry Cabral, Centro Hospitalar Universitário Lisboa Central, Lisbon, PRT

**Keywords:** intrahepatic cholangiocarcinoma, liver transplantation, locoregional treatments, neoadjuvant treatment, transarterial chemoembolization

## Abstract

Cholangiocarcinoma is a rare and heterogeneous disease that often requires multimodal treatment. The role of liver transplantation in these tumors has been controversial due to historically poor prognosis and higher recurrence rates. However, in recent years, scientific evidence has challenged this notion. We report the case of a 49-year-old woman with locally advanced intrahepatic cholangiocarcinoma. The therapeutic approach for this patient was complex, involving locoregional and systemic therapies. Despite the tumor's characteristics, namely, large size, multifocality, and vascular involvement, the good response to the treatment allowed a liver transplant 57 months after diagnosis.

## Introduction

Cholangiocarcinoma (CCA) is the second most common primary liver cancer, accounting for up to 15% of all hepatic malignancies [1]. These heterogeneous malignancies can arise anywhere in the biliary tree, classified based on their primary anatomical origin as intrahepatic CCA (iCCA), perihilar CCA, and distal CCA [1].

Most cases of CCA, approximately 70%, are sporadic. However, factors such as chronic inflammation of the biliary epithelium, bile stasis, obesity, metabolic syndrome, and high alcohol consumption are associated with an increased likelihood of developing this neoplasm [1,2].

Surgical treatment is the only potentially curative treatment for CCA patients, but only 15-35% of patients are eligible for this therapy due to the stage of the disease at the time of diagnosis [2]. However, even in patients who have undergone surgery, the results are disappointing, with a probability of cure of about 10% [3,4]. For locally advanced disease not amenable to upfront liver resection, neoadjuvant locoregional therapies such as hepatic arterial infusion (HAI) transarterial chemoembolization (TACE), transarterial radioembolization (TARE), thermal ablation and external-beam radiotherapy, and systemic treatment may be used with the aim of converting to the surgical approach [2-5].

Liver transplantation (LT) is another radical treatment option, but its applicability in CCA is controversial due to abysmal initial results with a high recurrence rate and lower survival benefit (two-year survival of around 30%) [4]. However, in recent years, the results of LT in CCA patients have improved with better patient selection and the use of neoadjuvant treatments [4-6]. Currently, two potential criteria for selecting iCCA patients for LT include very-early-stage tumors (<2 cm) with cirrhosis and locally advanced tumors with neoadjuvant chemotherapy [7].

Herein, we report the case of a 49-year-old female patient with unresectable iCCA. This patient underwent multiple cycles of chemotherapy and locoregional therapies over the years before undergoing a successful LT. She currently has an overall survival of 71 months and a disease-free survival of 16 months.

## Case presentation

**Figure 1:**
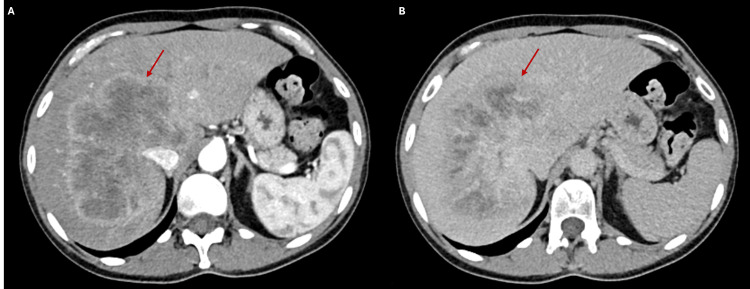
Initial CT showing a large liver lesion in the right lobe: (A) arterial phase and (B) delayed phase CT: computed tomography

A 49-year-old woman with no relevant personal and family medical history presented to a general medicine consultation with intense abdominal pain in the right upper quadrant during a Pilates class. She also reported asthenia and abdominal distension over a year. Abdominal ultrasound revealed a nodular lesion in the right hepatic lobe, measuring 10.7×8.4×11.1 cm. Blood tests showed elevated alkaline phosphatase (296 UI/L) and gamma-glutamyl transferase (179 UI/L). Thoracic-abdominal-pelvic computed tomography (CT) (Figure 1) and hepatic magnetic resonance imaging (MRI) confirmed a large liver lesion with suspicious characteristics, measuring 12×9 cm, occupying most of the right lobe and extending to segment IV of the left lobe. This lesion invaded the right branch of the portal vein and the right and middle suprahepatic veins. No other lesions were identified. The biopsy confirmed the diagnosis of iCCA. 

The patient was referred to our institution, and the clinical case was discussed at a multidisciplinary meeting. It was decided to perform TARE with yttrium-90 (Y90). Three months after TARE, imaging showed a slight increase in the size of the liver mass but with less contrast uptake. Therefore, the patient started chemotherapy with cisplatin 25 mg/m² and gemcitabine 1000 mg/m² administered intravenously on days 1 and 8 of a 21-day cycle. After nine cycles of chemotherapy, the lesion size reduced to 11.7×9.3 cm. A second TARE with Y90 was performed, and the patient continued treatment with gemcitabine 1000 mg/m² as monotherapy, with dosage reduction to improve tolerance. Given the sustained partial response to treatment, the patient underwent exploratory laparoscopy to assess the resectability of the tumor. Intraoperative ultrasound identified a suspected metastatic lesion in segment II. The patient resumed treatment with cisplatin and gemcitabine and, after eight cycles with a partial response, underwent another TARE with Y90. Re-assessment after the third TARE indicated a reduction in the primary tumor but an increase in the satellite lesions in segment II. The patient has then started chemotherapy with FOLFOX (folinic acid 50 mg on day 1, fluorouracil 400 mg/m^2^ bolus on day 1, fluorouracil 2400 mg/m^2^ on 46-hour continuous perfusion, and oxaliplatin 85 mg/m^2^ on day 1, 14-14 days) for eight cycles. Due to disease stability and poor treatment tolerance, the regimen was adjusted to the De Gramont (folinic acid 50 mg on day 1, fluorouracil 400 mg/m^2^ bolus on day 1, and fluorouracil 2400 mg/m^2^ on 46-hour continuous perfusion, 14-14 days).

**Figure 2:**
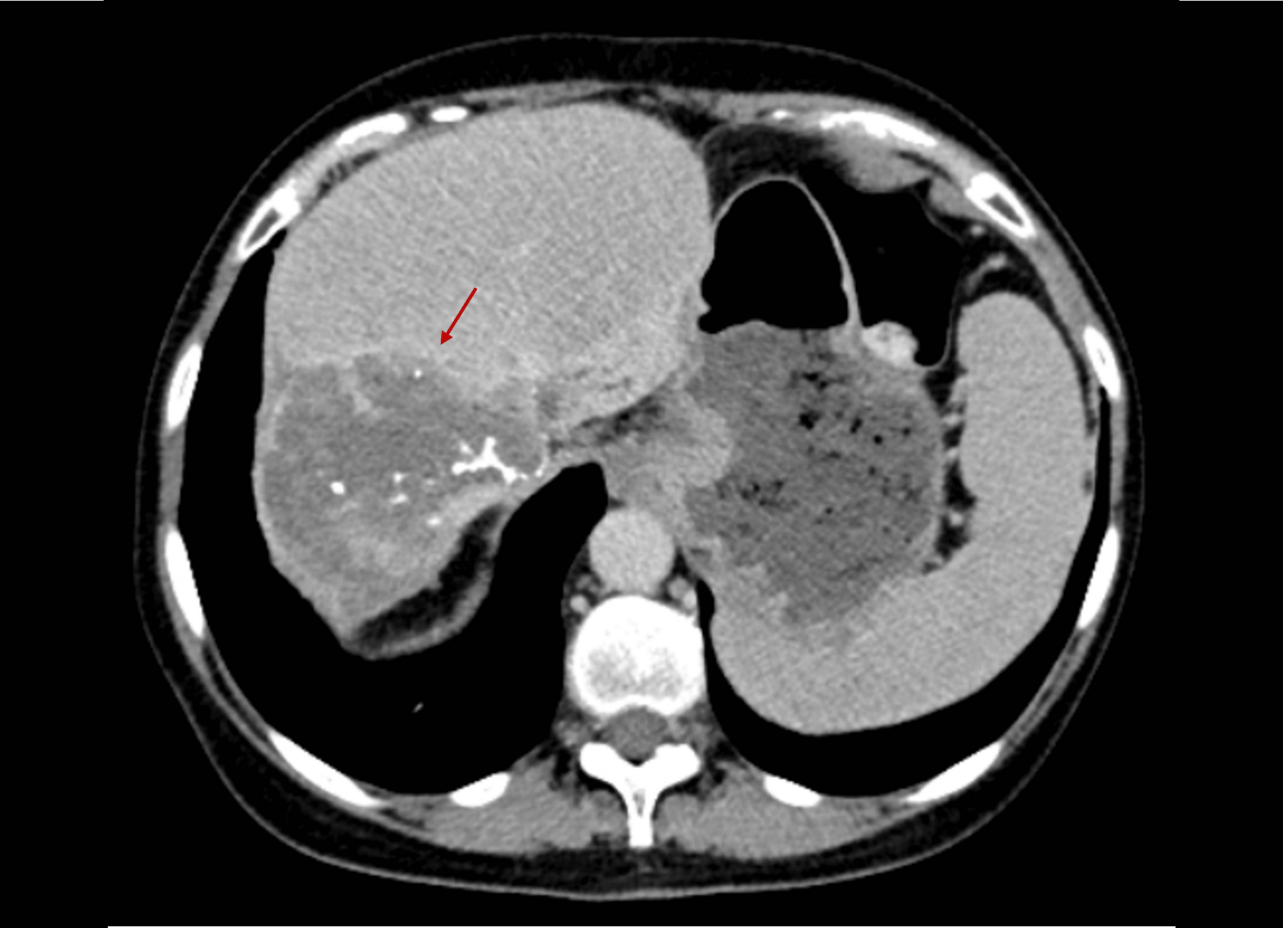
CT image before the patient was evaluated for liver transplantation (delayed phase) CT: computed tomography

Given the sustained response to treatment (Figure 2) and overall survival of more than 48 months, the patient was considered for LT. She underwent the procedure at another institution without complications. Histological examination of the explant confirmed intrahepatic small duct CCA with a mass-forming growth pattern and lymphovascular invasion, staged as ypT2N0. Following the transplant, the patient has undergone regular surveillance and has remained free of any disease manifestations for 16 months.

## Discussion

CCA is an aggressive malignancy that, although rare, is the second most common primary liver cancer after hepatocellular carcinoma (HCC) and represents 3% of all gastrointestinal malignancies [1,2]. The global trend over the past decades indicates an increase in the incidence of CCA [2,4]. More than 90,567 new cases were diagnosed worldwide in 2020 [6]. Despite significant advances in understanding molecular mechanisms, improved diagnostics, and developments in systemic and locoregional treatments, overall survival has not improved substantially [2]. CCA is a highly heterogeneous neoplasm, exhibiting a broad spectrum of clinical presentations, pathological features, and molecular characteristics [8]. These tumors are generally asymptomatic in the early stages, often resulting in late diagnosis. Their clinical presentation is non-specific and varies depending on the tumor's location, growth pattern, and stage of disease [8,9]. Morphologically, iCCA are further sub-classified into small- and large-duct variants. Small-duct-type iCCA, which was the case of our patient, occurs in the peripheral parts of the liver and typically remains asymptomatic until it reaches a large size. The most common symptoms are general malaise, nausea, abdominal pain in the right upper quadrant, and weight loss [8].

Liver resection is the cornerstone of treatment for these tumors, but only a minority of patients are able to undergo it [2,4]. An analysis of the Surveillance, Epidemiology, and End Results (SEER) database between 1983 and 2010 confirmed that only 15% of patients with iCCA underwent liver resection [4]. The tumor localization and the quantity and quality of the liver remaining after resection determine the feasibility of surgery [4]. Although liver resection is the only potentially curative treatment for CCA, survival outcomes are dismal, with a five-year overall survival of 25-40% and a risk of recurrence of over 60% [2,6]. A meta-analysis on curative resection of iCCA identified age, tumor size, multifocality, lymph node involvement, vascular invasion, and differentiation grade as predictors of recurrence and mortality [10]. Advances in surgical technique and neoadjuvant treatment have contributed to an increase in the number of hepatectomies. Neoadjuvant treatment contributes to the downstaging of the tumor, eradicates micrometastases, reduces the risk of recurrence, and improves patient selection by identifying those with rapid progression who will not benefit from surgical treatment [4,5].

A retrospective study assessing the efficacy of neoadjuvant chemotherapy revealed that among 74 patients with unresectable locally advanced iCCA, 53% underwent surgery following neoadjuvant treatment. Overall survival was comparable between patients who received chemotherapy and those who underwent surgery alone [11]. Another study demonstrated that, in patients with stage I-III iCCA, neoadjuvant chemotherapy was associated with a 22% reduced risk of death compared with upfront surgery followed by adjuvant chemotherapy [12].

Locoregional therapies are another treatment option for liver-confined, unresectable iCCA. These modalities include TACE, TARE, HAI, thermal ablation, and external-beam radiotherapy. Local therapies aim to promote local control and the downstaging of the tumor to resection, with few systemic effects [4,5]. TARE entails the transarterial delivery of radioactive glass or resin particles bound to Y90, which accumulates within the tumor [5]. In different studies, TARE has demonstrated improved survival compared to supportive care [3]. Additionally, combining TARE with chemotherapy (cisplatin plus gemcitabine) has been studied and shows promising results. In a phase 2 study, the combination of chemotherapy with TARE was addressed in 41 patients with locally unresectable iCCA. Nine patients (22%) showed downstage tumors and underwent surgery (eight patients R0 resection). The median overall survival was 22 months, and, at 46 months of follow-up after surgery, median relapse-free survival was not reached among patients who underwent liver resection [13].

LT is an alternative radical treatment for CCA. Nevertheless, due to historically high recurrence and low overall survival rates of these tumors, it is discouraged in most centers [4,5]. Becker et al., in a study involving 280 patients with iCCA who underwent LT, reported a five-year overall survival of 38% [14]. This figure is notably below the current clinical practice benchmark of achieving at least a 50-60% five-year overall survival post-transplant [2]. However, emerging research suggests that through accurate patient selection and the incorporation of neoadjuvant therapy, LT survival outcomes can be optimized. In a retrospective multicenter study involving patients who underwent LT with an occult iCCA discovered in the explant, findings revealed that "very early" iCCA (single tumor ≤2 cm) exhibited a higher five-year overall survival (65%) compared to "advanced" iCCA (single tumor >2 cm or multiple tumors) (45%). Moreover, the five-year cumulative risk of recurrence was significantly lower in "very early" iCCA (18%) compared to "advanced" iCCA (61%) [15]. A subgroup analysis of patients with "advanced" iCCA was divided into the "intermediate" stage (tumors ≤3 cm, not poorly differentiated) and the "advanced" stage. The five-year overall survival was 61% in the "intermediate" stage and 42% in the "advanced" stage.

Lunsford et al. demonstrated the role of neoadjuvant treatment. In a series of 12 patients with unresectable iCCA, six patients underwent LT after a minimum of six months of gemcitabine-based chemotherapy with radiographic response or stability. The five-year overall survival rate was 83.3%, while the five-year recurrence-free survival rate was 50% [16]. Another study with 32 patients with locally advanced, unresectable, iCCA showed that 18 patients underwent LT after neoadjuvant chemotherapy. Survival at five years was 48% [17]. According to the literature, some factors such as tumor size, multifocality, vascular invasion, and poor tumor differentiation are predictors of worse outcomes with LT [6]. Therefore, despite limited scientific evidence due to the absence of prospective randomized trials, two potential selection criteria for patients with iCCA undergoing LT are very-early-stage tumors arising in the context of cirrhosis and locally advanced tumors with response after neoadjuvant chemotherapy [4-7].

In our case, the patient had locally advanced iCCA with multifocal disease and no extrahepatic involvement. The decision to perform an LT was influenced by her age, good performance status, and long response to treatments over the years. Despite risk factors such as a large, multifocal tumor with vascular involvement, the patient has remained disease-free for 16 months.

## Conclusions

The case presented highlights the challenges in treating locally advanced iCCA and the evolving role of LT in this setting. Despite historically poor prognosis and high recurrence rates, advancements in neoadjuvant therapies and patient selection criteria have shown promising results in improving outcomes for patients with unresectable CCA. Prospective randomized studies are needed to validate the role of LT in iCCA. Furthermore, it is crucial to establish precise patient selection criteria to discern individuals who can truly benefit from this therapeutic approach.

The treatment of CCA is complex and requires multiple treatment modalities, making it essential to discuss cases in a multidisciplinary meeting. Given the disease's heterogeneity and inter-individual variability, an individualized approach is necessary for each patient, grounded in scientific evidence.
